# Epitope mapping of anti-PGRMC1 antibodies reveals the non-conventional membrane topology of PGRMC1 on the cell surface

**DOI:** 10.1038/s41598-018-37441-6

**Published:** 2019-01-24

**Authors:** Ji Yea Kim, So Young Kim, Hong Seo Choi, Sungkwan An, Chun Jeih Ryu

**Affiliations:** 1 Institute of Anticancer Medicine Development, Department of Integrative Bioscience and Biotechnology, Sejong University, Seoul, Republic of Korea; 2 Research Institute for Molecular-Targeted Drugs, Department of Cosmetic Engineering, Konkuk University, Seoul, Republic of Korea; 3 Present Address: Research Institute for Molecular-Targeted Drugs, Department of Cosmetic Engineering, Konkuk University, Seoul, Republic of Korea

## Abstract

Progesterone receptor membrane component1 (PGRMC1) is a heme-binding protein involved in cancers and Alzheimer’s disease. PGRMC1 consists of a short N-terminal extracellular or luminal domain, a single membrane-spanning domain, and a long cytoplasmic domain. Previously, we generated two monoclonal antibodies (MAbs) 108-B6 and 4A68 that recognize cell surface-expressed PGRMC1 (csPGRMC1) on human pluripotent stem cells and some cancer cells. In this study, flow cytometric analysis found that an anti-PGRMC1 antibody recognizing the N-terminus of PGRMC1 could not bind to csPGRMC1 on cancer cells, and 108-B6 and 4A68 binding to csPGRMC1 was inhibited by trypsin treatment, suggesting that the epitopes of 108-B6 and 4A68 are trypsin-sensitive. To examine the epitope specificity of 108-B6 and 4A68, glutathione-S-transferase (GST)-fused PGRMC1 mutants were screened to identify the epitopes targeted by the antibodies. The result showed that 108-B6 and 4A68 recognized C-terminal residues 183–195 and 171–182, respectively, of PGRMC1, where trypsin-sensitive sites are located. A polyclonal anti-PGRMC1 antibody raised against the C-terminus of PGRMC1 could also recognized csPGRMC1 in a trypsin-sensitive manner, suggesting that the C-terminus of csPGRMC1 is exposed on the cell surface. This finding reveals that csPGRMC1 has a non-conventional plasma membrane topology, which is different from that of intracellular PGRMC1.

## Introduction

Progesterone receptor membrane component 1 (PGRMC1) is a multifunctional protein with a C-terminal cytochrome *b*_5_ domain^1^. PGRMC1 is highly expressed in multiple types of cancer, and represents a proliferation marker for various cancer cells^1–4^. PGRMC1 also increases the neuronal toxicity of amyloid β-peptides through binding to amyloid β oligomer in Alzheimer’s disease^5,6^. PGRMC1 is also involved in diverse biological functions, such as regulation of cytochrome P450, progesterone signaling, vesicle trafficking, steroidogenesis, cell cycle regulation, anchorage-independent growth, invasive growth, angiogenesis, hypoxic biology, and autophagy promotion^1,7^. PGRMC1 consists of a short N-terminal luminal or extracellular domain, a single membrane-spanning domain, and a long cytoplasmic domain^8–11^. Many studies have shown that PGRMC1 is localized at various subcellular compartments, such as endoplasmic reticulum, Golgi apparatus, plasma membrane, inner acrosomal membrane, nucleus, nucleolus, and mitochondria^9,12–15^. PGRMC1 regulates cell proliferation and apoptosis in granulosa and luteal cells via interaction between its cytoplasmic cytochrome *b*_5_ binding domain (amino acids 70–130) and plasminogen activator inhibitor RNA-binding protein-1 (PAIR-BP1)^10,16^. PGRMC1 can also interact or associate with other binding partners including epidermal growth factor receptor (EGFR)^17^, glucagon-like peptide-1 receptor (GLP-1R)^18^, insulin receptor^19^, glucose channels^19^, membrane progesterone receptor (mPRα/PAQR7)^20^, and P450 proteins^21,22^. A recent study revealed that the heme-mediated dimerization of adjacent PGRMC1 monomers in the cytoplasmic side leads PGRMC1 to interact with cytochromes P450 and EGFR, causing enhanced proliferation, anti-apoptosis, and chemoresistance of cancer cells^22^. However, the conclusion is challenged with some observations and thoughts that tyrosine 113 phosphorylation of PGRMC1 is required for membrane trafficking to co-localize PGRMC1 and EGFR^23^, and the cytoplasmic domain of PGRMC1 is located on the luminal side of microsomes in A549 cells^17^.

Previously, we generated a panel of murine monoclonal antibodies (MAbs) against the surface molecules on undifferentiated human pluripotent stem cells (hPSCs) by using a modified decoy immunization strategy^24^. Subsequent studies showed that 108-B6 and 4A68, two of the MAbs, bind to cell surface expressed-PGRMC1 (csPGRMC1) on hPSCs and some cancer cells^25^. PGRMC1 knockdown approach further revealed that PGRMC1 suppresses the p53 and Wnt/β-catenin pathways to promote hPSC self-renewal^25^. Meanwhile, flow cytometric analysis found that an anti-PGRMC1 antibody recognizing the N-terminal domain (residues 1–46) of PGRMC1 was not able to bind to csPGRMC1 on cancer cells, although it was able to recognize intracellular PGRMC1 in saponin-treated cells. Flow cytometric analysis also showed that 108-B6 and 4A68 binding to csPGRMC1 was inhibited by trypsin treatment, suggesting that the epitopes of 108-B6 and 4A68 is outside the N-terminal domain and have trypsin-sensitive sites within them. This observation led us to investigate the epitope of two MAbs on PGRMC1. The results revealed that 108-B6 recognized C-terminal residues 183–195 of PGRMC1, and 4A68 recognized C-terminal residues 171–182 of PGRMC1, where putative trypsin-sensitive sites are located. Thus, this finding reveals that the C-terminal domain of PGRMC1 is exposed on the cell surface, instead of the N-terminal domain of PGRMC1. A polyclonal anti-PGRMC1 raised against the C-terminal domain of PGRMC1 also recognized csPGRMC1, supporting that the C-terminal domain of PGRMC1 is exposed on the cell surface. Thus, epitope analysis of PGRMC1 antibodies reveals that csPGRMC1 has a different membrane topology from that of intracellular PGRMC1.

## Results

### Monoclonal antibody against the N-terminus of PGRMC1 is not able to recognize csPGRMC1, whereas 108-B6 and 4A68 is able to recognize csPGRMC1

**Figure 1:**
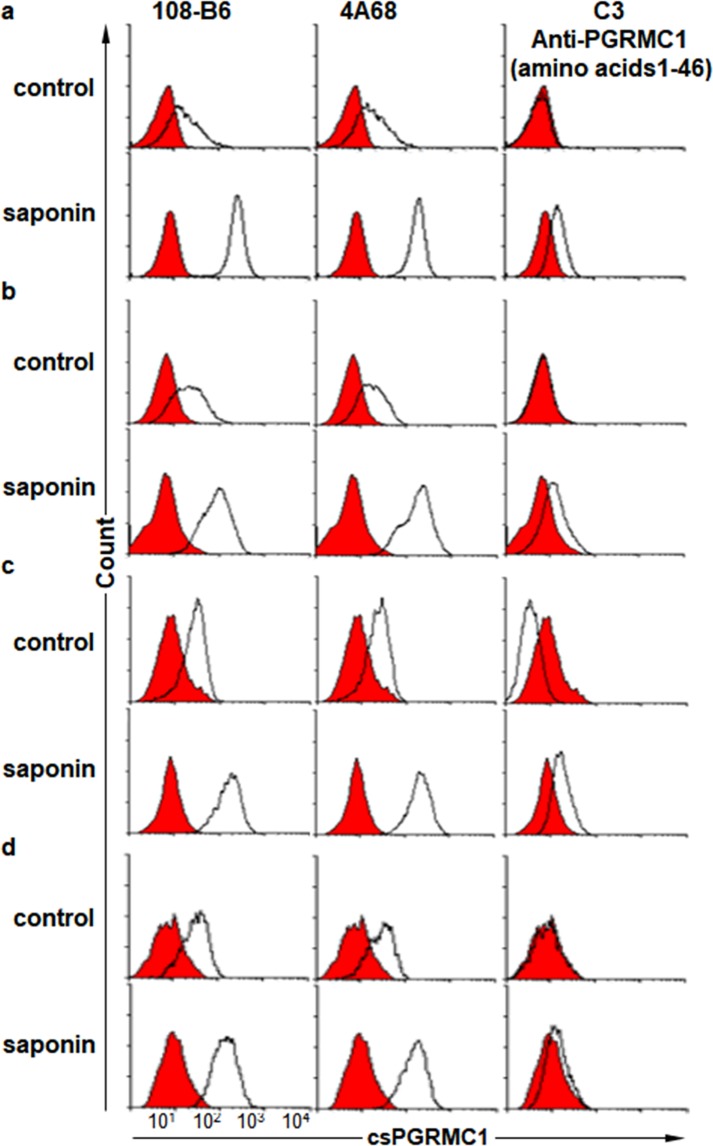
Monoclonal antibody C3 against the N-terminus of PGRMC1 is not able to recognize csPGRMC1, whereas 108-B6 and 4A68 is able to recognize csPGRMC1. (**a**–**d**) Flow cytometric analysis of H9 (**a**), NT-2/D1 (**b**), HEK293T (**c**), and HepG2 (**d**) with 108-B6, 4A68, and an anti-PGRMC1 antibody (C-3) in the absence (control) or presence of saponin (saponin). C3 is a monoclonal anti-PGRMC1 raised against the N-terminal residues 1–46. Red populations indicate fluorescence-conjugated secondary antibody staining as controls.

In the previous study, we generated two MAbs, 108-B6 and 4A68, against cell surface molecules on hPSCs, and found that both MAbs recognize csPGRMC1 on hPSCs and some cancer cells^24,25^. Many previous studies have shown that PGRMC1 has a short N-terminal luminal or extracellular domain (residues 1–20), a single membrane-spanning domain (residues 21–42), and a much longer cytoplasmic domain (residues 43–195)^8–11,26^. Therefore, we expected that 108-B6 and 4A68 recognized epitopes on the short N-terminal extracellular domain because they were able to recognize csPGRMC1. However, a commercially available antibody (C3) raised against the N-terminal domain (amino acids 1–46) of PGRMC1 was not able to recognize csPGRMC1 at all on the surface of H9, NT-2/D1, HEK293T and HepG2 cells while 108-B6 and 4A68 were able to recognize csPGRMC1 on the cell surface of all cells (Fig. 1a–d, upper panels). When PGRMC1 protein was immunoprecipitated with 108-B6 and 4A68, immnoprecipitated proteins were readily detected with C3, indicating that C3 is able to recognize 108-B6- and 4A68-reactive PGRMC1 protein in Western blot analysis (Supplementary Fig. 1). To further analyze whether the C3 antibody is functional in intracellular flow cytometric analysis, cells were analyzed in the presence of saponin detergent. The binding activity of 108-B6 and 4A68 was increased in all cells with saponin treatment, suggesting the increased accessibility of intracellular PGRMC1 (Fig. 1a–d, lower panels). The C3 antibody was also able to recognize intracellular PGRMC1 in the saponin-treated cells (Fig. 1a–d, lower panels). The results suggest that the C3 antibody is functional and is able to recognize the N-terminal domain of PGRMC1 when cells are permeabilized. Therefore, it is possible to speculate that the N-terminal domain of PGRMC1 may be located in the cytoplasmic side of H9, NT-2/D1, HEK293T and HepG2 cells.

### Trypsin treatment drastically abolishes binding reactivity of 108-B6 and 4A68 to csPGRMC1

**Figure 2:**
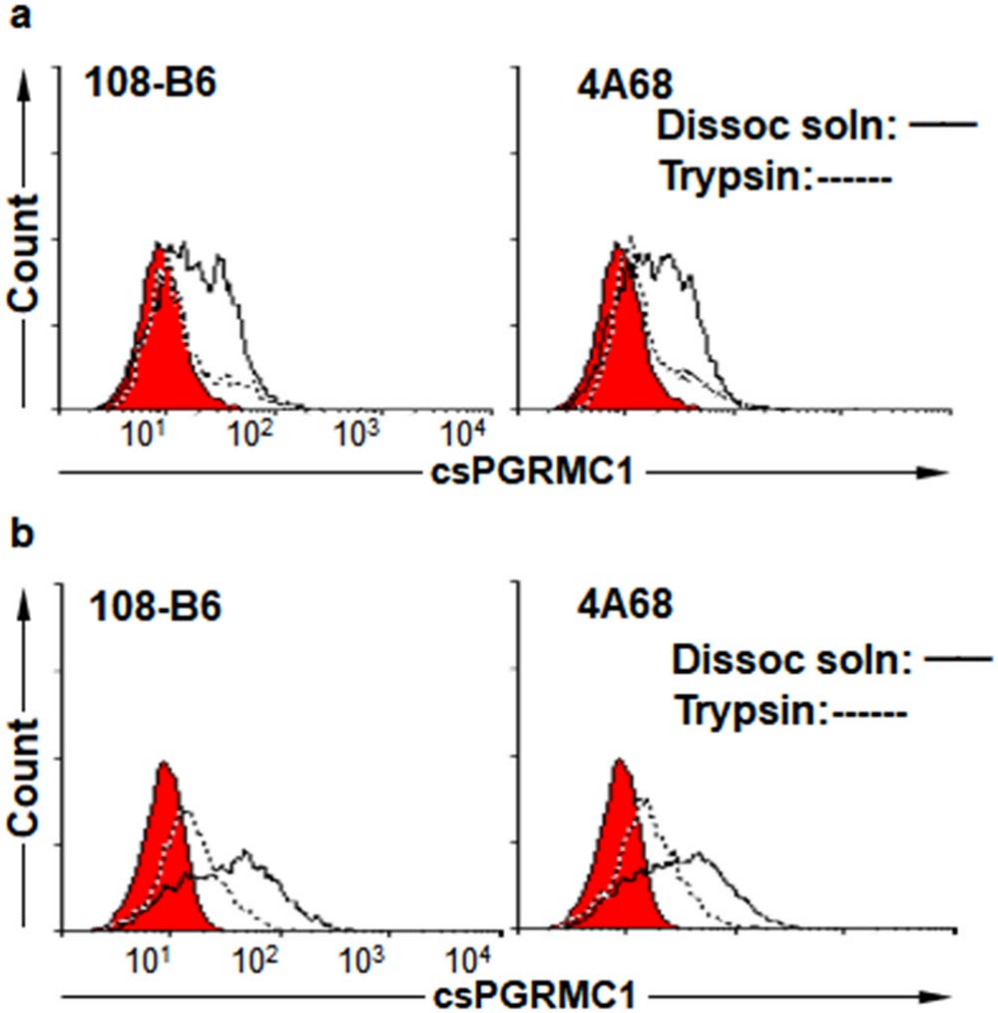
Trypsin treatment drastically abolishes binding reactivity of 108-B6 and 4A68 to csPGRMC1. (**a**) Flow cytometric analysis of NT-2/D1 cells with 108-B6 and 4A68 after detachment of cells with trypsin or enzyme-free dissociation solution. Red populations indicate fluorescence-conjugated secondary antibody staining as controls. (**b**) Flow cytometric analysis of NCCIT cells with 108-B6 and 4A68 after detachment of cells with trypsin or enzyme-free dissociation solution.

Meanwhile, flow cytometric analysis showed that binding reactivity of 108-B6 and 4A68 to cancer cells was variable depending on detachment method. To figure out how the detachment method affected the binding reactivity of two MAbs to NT-2/D1 cells, cells were detached with trypsin or enzyme-free dissociation solution, and propidium iodide (PI)-negative live cells were subjected to flow cytometric analysis. Interestingly, trypsin treatment abolished the binding reactivity of two MAbs drastically, as compared with dissociation solution (Fig. 2a). The similar result was also observed with NCCIT cells, another human embryonal carcinoma cell line (Fig. 2b). The results suggest that the epitopes of 108-B6 and 4A68 is cell surface-exposed and trypsin-sensitive. Sequence analysis showed that there is no putative trypsin-sensitive site on the N-terminal domain of PGRMC1, further suggesting that the other domain of PGRMC1 is exposed on the extracellular side, instead of the N-terminal domain of PGRMC1.

### Comparison of amino acid sequences of 108-B6 and 4A68 variable regions

Although 108-B6 and 4A68 recognized the same csPGRMC1 protein, they showed slight different binding reactivity to U87-MG, NCI-H522 and A549^25^, suggesting that they recognize different binding epitopes on csPGRMC1 protein. The result also suggests that 108-B6 and 4A68 are different antibodies with different complementarity determining regions (CDRs). To compare amino acid sequence of 108-B6 and 4A68 variable regions, the heavy- and light-chain variable regions of two MAbs were cloned and sequenced by using universal degenerate primers^27^. The complementarity determining regions (CDRs) of 108-B6 and 4A68 were identified by using information of Abysis database (http://www.bioinf.org.uk). Although 108-B6 and 4A68 recognized the same PGRMC1 protein, sequence analysis showed that the CDRs of heavy and light chains of two MAbs were quite different to each other (Supplementary Fig. 2). Especially, the lengths and sequences of heavy chain CDR3s were completely different from each other, suggesting that they may recognize different epitopes on csPGRMC1.

### Fine epitope mapping of 108-B6 and 4A68 antibodies

**Figure 3:**
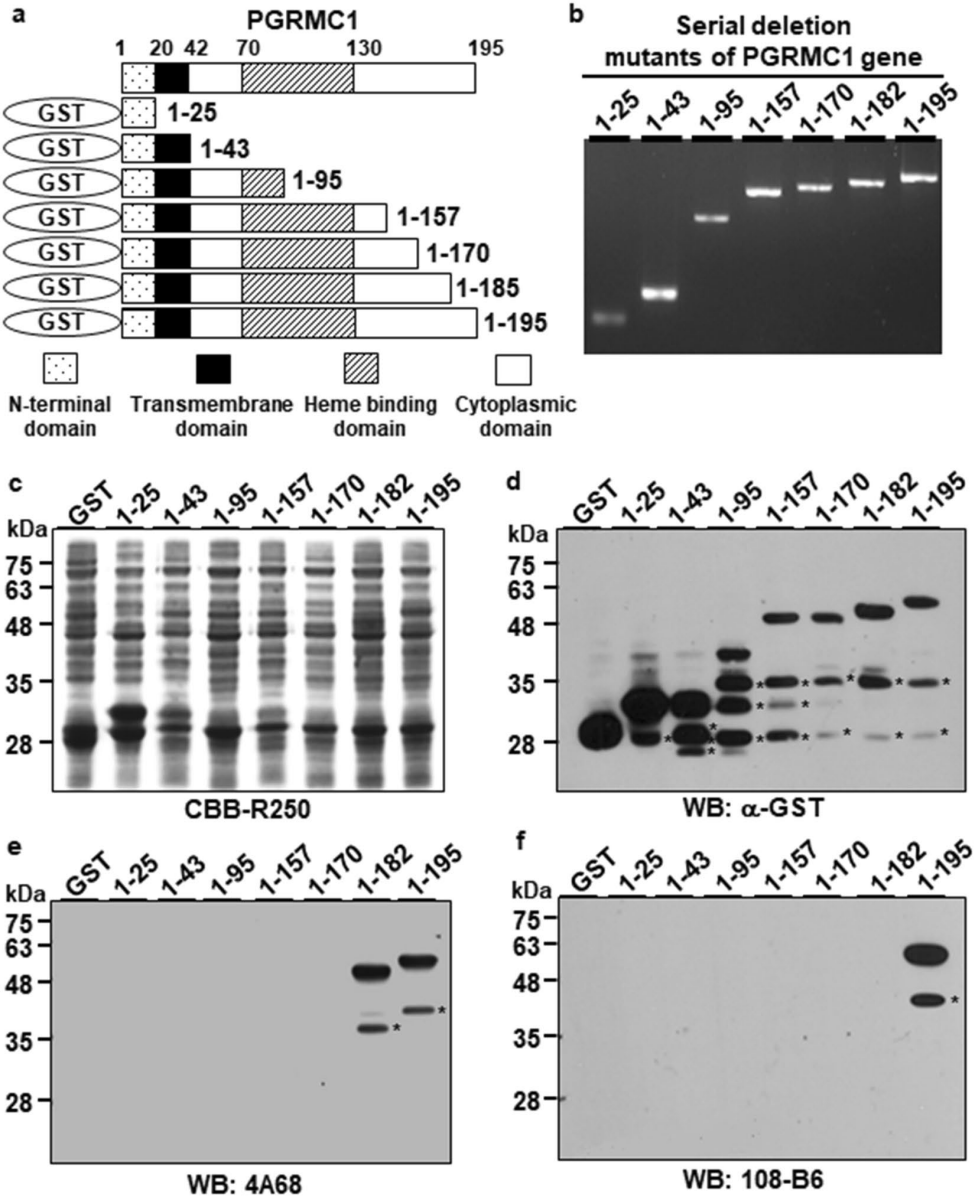
Fine epitope mapping of 108-B6 and 4A68 antibodies. (**a**) Schematic diagram of recombinant PGRMC1 fragments (residues 1–25, 1–43, 1–95, 1–157, 1–170, 1–182 and 1–195) used in this study. (**b**) A series of depletion mutants of PGRMC1 gene were synthesized and separated by agarose gel electrophoresis. The deletion mutants of PGRMC1 genes were detected by ethidium bromide staining. (**c**) Individual fusion proteins were expressed in *E. coli* DH5α as fusion proteins with GST tag at the N-terminus, and stained with CBB R-250 after SDS-PAGE. (**d**,**f**) Western blot analysis of GST-PGRMC1 fusion proteins with anti-GST (**d**), 4A68 (**e**), and 108-B6 (**f**) antibodies. The asterisks indicate partial degradation products of GST-PGRMC1 fusion proteins.

To examine the epitopes of 108-B6 and 4A68 on csPGRMC1 protein, a series of deletion mutants of PGRMC1 gene were generated as shown in Fig. 3a, and synthesized by PCR (Fig. 3b). Each cDNA was cloned into pGEX4T-2 cloning vector to tag glutathione-S-transferase (GST) gene and the fusion constructs were introduced into *E. coli* DH5α. The expression of a series of GST-fused PGRMC1 proteins were induced by isoprophyl-β-D-thiogalactopyranoside (IPTG) and judged by Coomassie Brilliant Blue (CBB) staining and Western blot analysis with anti-GST antibody, which showed the expression of expected sizes of GST-PGRMC1 fusion proteins, although the partially degraded forms of the serial deletion mutants of GST-PGRMC1 fusion proteins were also detected below the main GST-PGRMC1 fusion proteins (Fig. 3c,d). The same lysates were then subjected to Western blot analysis with 4A68 and 108-B6 (Fig. 3e,f). 4A68 recognized the wild-type PGRMC1 (residues 1–195) and one of deletion mutants (residues 1–182) but did not recognize the other deletion mutants (residues 1–25, 1–43, 1–95, 1–157 and 1–170), indicating that 4A68 recognizes the linear epitopes located between residues 171–182 of PGRMC1. 108-B6 recognized only the wild-type PGRMC1 (residues 1–195), but did not recognize any other deletion mutants (residues 1–25, 1–43, 1–95, 1–157, 1–170 and 1–182), indicating that 108-B6 recognizes the linear epitopes located between residues 183–195 of PGRMC1. The results suggest that the antigen binding sites of 4A68 and 108-B6 at least require residues 171–182 and 183–195, respectively, of PGRMC1 protein, although it could not exclude other residues from the body of the folded PGRMC1 protein. Generally, antibodies only access and recognize cell surface-exposed epitopes on live cells. Therefore, the results suggest that the epitope regions of 108-B6 and 4A68 are exposed on the cell surface.

### Polyclonal antibody against the C-terminus of PGRMC1 is able to recognize csPGRMC1

**Figure 4:**
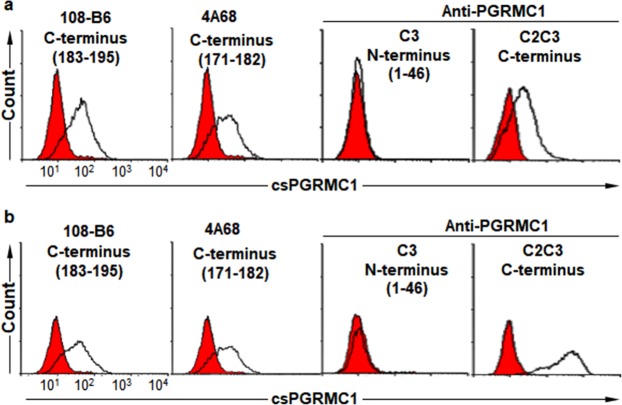
Polyclonal antibody against the C-terminus of PGRMC1 is able to recognize csPGRMC1. Flow cytometric analysis of NT-2/D1 and H9 hPSCs with 108-B6, 4A68, and anti-PGRMC1 antibodies (C3 and C2C3) after detachment of NT-2/D1 (**a**) and H9 hPSCs (**b**) with dissociation solution. C2C3 is a rabbit polyclonal anti-PGRMC1 raised against the C-terminal region of PGRMC1. Red populations indicate fluorescence-conjugated secondary antibody staining as controls.

Residues 171–182 and 183–195, recognized by 4A68 and 108-B6, respectively, belong to the last C-terminal part of PGRMC1. Therefore, the present results strongly suggest that the C-terminal domain of PGRMC1 is exposed on the extracellular side, although previous studies have shown that an N-terminal domain of PGRMC1 is exposed on the cell surface^8–11^. Therefore, another commercially available polyclonal anti-PGRMC1 antibody (C2C3) raised against the C-terminal domain of PGRMC1 was also included in flow cytometric analysis. As expected, C2C3 was able to recognize csPGRMC1 on NT-2/D1 and H9 hPSCs while C3 was not able to recognize csPGRMC1 (Fig. 4). The same results were also obtained with A549 cells (Supplementary Fig. 3). Trypsin treatment decreased C2C3 binding to csPGRMC1 on A549 cells as well, suggesting that the epitope of C2C3 also contains trypsin-sensitive sites as the epitopes of 4A68 and 108-B6. Taken together, the results suggest again that the C-terminal domain of PGRMC1 is exposed on the cell surface, instead of the N-terminal domain of PGRMC1.

## Discussion

**Figure 5:**
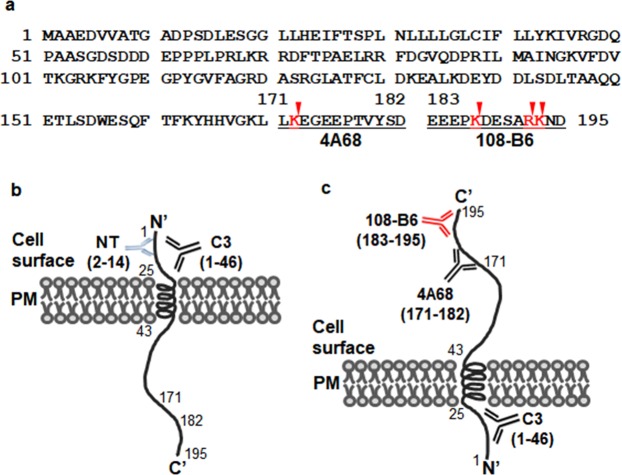
Proposed model for the plasma membrane topology of csPGRMC1. (**a**) Amino acid sequences of PGRMC1. The epitopes determined in the present study are underlined. Putative trypsin sensitive sites on the epitopes are marked by arrow heads. (**b**) The membrane topology of csPGRMC1 based on the previous studies^8–11,26^. NT and C3 represent antibodies raised against the N-terminal domain of PGRMC1. (**c**) Newly proposed plasma membrane topology of csPGRMC1 based on the present study. The antigen binding sites of 4A68 and 108-B6 at least require residues 171–182 and 183–195, respectively, of PGRMC1 protein, although it could not exclude other residues from the body of the folded PGRMC1 protein.

The present study found that two MAbs, 108-B6 and 4A68, recognized csPGRMC1 (Fig. 1), and their epitopes were located between residues 183–195, 171–182, respectively, of PGRMC1 (Fig. 3d–f). Generally, antibodies can access and recognize cell surface exposed-epitopes, and the residues 171–195 are located in the last C-terminal domain of PGRMC1. Therefore, the present results suggest that the C-terminal domain of csPGRMC1 is exposed on the cell surface. csPGRMC1 was also recognized by C2C3, a commercially available polyclonal anti-PGRMC1 antibody raised against the C-terminal domain of PGRMC1 (Fig. 4 and Supplementary Fig. 3). Furthermore, C3, another monoclonal anti-PGRMC1 antibody generated against the N-terminal domain (residues 1–46) of PGRMC1, was not able to detect csPGRMC1, although it was able to recognize the N-terminal domain of PGRMC1 after cell permeabilization with saponin (Fig. 1a–d). The results suggest that the C-terminal domain of PGRMC1 is exposed on the cell surface, but the N-terminal domain of PGRMC1 is not exposed on the cell surface. The binding reactivity of 108-B6 and 4A68 to NT-2/D1, NCCIT, and A549 cells was decreased with trypsin treatment. Sequence analysis revealed that trypsin-sensitive sites are present in the epitopes of 108-B6 and 4A68 (just behind residues 172, 187, 192 and 193) (Fig. 5a). Therefore, it is highly likely that decreased reactivity of 108-B6, 4A68 and C2C3 to csPGRMC1 on trypsin-treated cells is due to the cleavages of their epitopes by trypsin. Thus, the results suggest that the C-terminal domain (residues 171–195) of csPGRMC1 is at least exposed on the extracellular side. However, it has been known that PGRMC1 consists of a short N-terminal extracellular domain, a single membrane-spanning domain, and a long cytoplasmic domain (Fig. 5b)^8–11^. Therefore, characterizing the epitopes of 108-B6, 4A68 and C3 antibodies revealed the presence of the non-conventional reverse topology of csPGRMC1 on the surface of hPSCs and some cancer cells. A proposed model for the non-conventional plasma membrane topology of csPGRMC1 is therefore presented (Fig. 5c).

PGRMC1 is mainly localized in the endoplasmic reticulum, mitochondria, nucleus membrane, and nucleolus in multiple cancer cells^1,11^. Some studies have also shown that PGRMC1 is expressed on the surface of cancer and neuronal cells^5,17,28–30^. PGMRC1 consists of a short N-terminal extracellular domain, a single membrane-spanning domain, and a much longer cytoplasmic domain^8–11,26,31^ (Fig. 5b). We found that 108-B6- and 4A68-reactive csPGRMC1 was expressed on hPSCs and a few cancer cell lines^25^. In the very beginning, we expected that 108-B6 and 4A68 recognized the short N-terminal extracellular domain of PGRMC1 in hPSCs and cancer cells, because the cytoplasmic side of membrane proteins remains on the cytoplasmic side while the luminal side of membrane proteins is exposed on the cell surface even during the process of cell surface translocation^32,33^. Contrary to our expectation, epitope mapping of 108-B6 and 4A68 reveals that the C-terminal domain of PGMRC1 is exposed on the cell surface, suggesting an unexpected membrane topology of PGRMC1 on the cell surface. A previous study demonstrated that PGRMC1 is not affected by proteinase K digestion of a microsomal fraction without detergent in A549 cells^17^, suggesting a luminal orientation for the cytoplasmic domain of PGRMC1 in A549 cells. The result also suggests that the C-terminal domain of PGRMC1 would be exposed on the cell surface of A549 cells during the process of cell surface translocation. In this study, we showed that the C-terminal domain of PGRMC1 is exposed on the cell surface of A549 cells (Supplementary Fig. 3), which is consistent with the previous prediction^17^. The other studies have also observed extracellular PGRMC1 on the surface of neurons^5,34^. The studies also suggest that the C-terminus of PGRMC1 is located extracellularly in neurons, which is consistent with the present finding.

The present study suggests the existence of the opposite membrane topology of PGRMC1 on the cell surface. Based on the present study, however, it could not exclude the possibility that both the N- and C-terminus of PGRMC1 could be simultaneously present on the extracellular surface, where the extracellular N-terminus could interact with a natural extracellular ligand and be inaccessible for C3 binding, while the C-terminus could interact with 108-B6, 4A68 and C2C3. The present findings could also be harmonized by the multiple topologies of PGRMC1. Multiple or dynamic topologies are found in some membrane proteins, such as ductin, cystic fibrosis transmembrane conductance regulator, aquaporin-1 and P-glycoprotein^35–41^. Actually, Cahill and Medlock also suggested the possibility of alternative post-translational topologies of PGRMC1^11^. However, the possibility of multiple topologies of csPGRMC1 seems to be low because C3 recognizing the N-terminal domain of PGRMC1 was not able to recognize csPGRMC1 on hPSCs and some cancer cells (Figs 1 and 4). As described in many literatures^1,11,42,43^, the major function of PGRMC1 is based on the interaction between the cytoplasmic C-terminal domain of PGRMC1 and intracellular factors. Therefore, the biological significance of the opposite membrane topology of PGRMC1 will be the next interesting research subject.

## Methods

### Cell Culture

H9 hPSCs were cultured on the irradiated mouse embryonic fibroblast (MEF) feeder cells in DMEM/F12 medium (WelGene, Daegu, Korea), supplemented with 20% serum replacement (Invitrogen, Seoul, Korea), 0.1 mM 2-mercaptoethanol, 1% non-essential amino acids, 32 mM sodium bicarbonate, and 4 ng/ml basic fibroblast growth factor (bFGF) (PeproTech, Rocky Hill, NJ)^24,44^. Human embryonal carcinoma cell lines NT-2/D1 and NCCIT were cultured according to the instructions provided by American Type Culture Collection (ATCC, Manassas, VA). The non-small cell lung carcinoma cell line A549 was obtained from ATCC and maintained according to the protocol provided by the supplier. HepG2 was purchased from Korean Cell Line Bank (Seoul, Korea). Hybridomas 108-B6 and 4A68 were cultured at 5% CO_2_, 37 °C in DMEM (WelGene) supplemented with 10% fetal bovine serum (WelGene).

### Antibody Purification

MAbs were purified from the culture supernatant of hybridomas by Protein G-Agarose column chromatography as described previously^24,45^.

### Flow cytometry

H9, NT-2/D1, HEK293T, HepG2, NCCIT and A549 cells were harvested as single cell suspensions using trypsin/EDTA (Welgene, Daegu, Korea) solution or enzyme-free dissociation solution (Millipore, Billerica, MA). Detached cells were immediately resuspended in PBA (1% bovine serum albumin, 0.02% NaN_3_ in phosphate buffered saline (PBS), pH7.4) and incubated for 20 min at 4 °C with 108-B6, 4A68, rabbit polyclonal-anti-PGRMC1 antibody (C2C3, GeneTex, Irvine CA), and mouse monoclonal anti-PGRMC1 (C3, Santa Cruz Biotechnologies, Santa Cruz, CA). The cells were then further incubated with fluorescein isothiocyanate (FITC)-conjugated anti-mouse IgG or FITC-conjugated anti-rabbit IgG (BD Biosciences, Seoul, Korea). After washing, propidium iodide (PI)-negative live cells were analyzed for antibody binding using FACS Calibur and Cell Quest software (BD Biosciences). For intracellular flow cytometric analysis, cells were fixed in 2% paraformaldehyde (PFA) in PBS (pH 7.4), permeabilized in 0.5% saponin (Sigma-Aldrich, Seoul, Korea) in PBA for 15 min at 4 °C, and then washed twice with PBA. The cells were incubated with 108-B6, 4A68 or C-3 antibodies for 15 min at 4 °C, subsequently incubated with FITC-conjugated mouse IgG (BD Biosciences) for 15 min at 4 °C, and analyzed for the antibody binding using FACS Calibur (BD Biosciences) and Cell Quest software (BD Biosciences).

### Western blot analysis

Total extracts of various cells were obtained after lysis for 30 min at 4  °C in RIPA buffer (150 mM NaCl, 1% NP-40, 0.5% Doxycholic acid, 0.1% SDS, 50 mM Tris-HCl (pH 7.4)). Protein samples were fractionated by sodium dodecyl-sulfate-polyacrylamide gel electrophoresis (SDS-PAGE) on a 12% polyacrylamide gel under denaturing conditions and transferred to a nitrocellulose membrane. The membrane was blocked with 5% skim milk in PBST (PBS containing 0.1% Tween 20) at room temperature (RT) for 2 hrs. After washing with PBST, the membrane was incubated with various primary antibodies at RT for 1 hr, followed by horseradish peroxidase (HRP)-conjugated anti-mouse IgG (Millipore). The stained bands were visualized by using ECL (Animal Genetics, Gyeonggi-do, Korea) detection reagent.

### cDNA cloning and sequencing

Total RNAs were extracted from 108-B6 and 4A68 hybridoma cells with RNA iso plus reagent (TaKaRa, Otsu, Japan) according to the supplier’s protocol. cDNAs were generated from total RNAs by Prime Script RT Master Mix (TaKaRa), and used for polymerase chain reaction (PCR) amplification to obtain the coding regions of variable heavy and light chains of two MAbs by standard reverse transcriptase (RT)-PCR using specific primers as described previously^27^. The amplified gene segments were subcloned into pBluescript cloning vector and used to transform DH5α bacterial cells. Selected plasmids were sequenced using the M13 primers (Cosmo Genetech, Seoul, Korea).

### Preparation and induction of GST-fusion protein

Serially truncated PGRMC1 proteins were expressed as fusion proteins with Glutathione-S-transferase (GST) proteins. The coding sequences of serially truncated and whole PGRMC1 genes were synthesized by PCR from the pCMV-SPORT6-PGRMC1 plasmid using 5′-primer and various 3′-primers and subcloned into the EcoRI/SalI sites of pGEX4T-2 (GE Healthcare, Seoul, Korea) to yield the expression plasmids. All primer sequences are listed in Table 1. Each expression plasmid was confirmed by DNA sequencing, and introduced into *E. coli* DH5α cells to express the GST-PGRMC1 fusion proteins. The expression of the fusion proteins was induced by 0.1 mM IPTG at 32 °C for 3 hrs. The induced bacterial cells were washed with pre-chilled PBS (pH 7.4), incubated with acetone on ice for 5 min, and lysed in 1% SDS supplemented with 100 μg/ml phenylmethanesulfonyl fluoride for 2 min at room temperature (RT). Proteins were clarified by centrifugation, and their concentration was measured by bicinchoninic assay (Thermo Scientific, Seoul, Korea). The cell lysates were subjected to 12% SDS-PAGE, stained with CBB R-250, and analyzed by western blot analysis as described above.

**Table 1:** Primer sequences for serial deletion mutants of PGRMC1.

PGRMC1 cDNA	Sequence (5′– 3′)
PGRMC1 sense	5′- CTCGAATTCTCATGGCTGCCGAG-3′
(1–25) antisense	5′-CGGCTCGAGTTAAATCTCATGCAGCAG-3′
(1–43) antisense	5′-GCGCTCGAGTTAGTAGAGCAGGAAGAT-3′
(1–95) antisense	5′-TCTCTCGAGTTAAAAGACCCCATACGG-3′
(1–157) antisense	5′-AGTCTCGAGTTACTCCCAGTCACT-3′
(1–170) antisense	5′- CCCCTCGAGTTACAGTTTGCCCAC-3′
(1–182) antisense	5′- TGGCTCGAGTTAATCTGAGTACAC-3′
(1–195) antisense	5′-CCCCTCGAGTTAATCATTTTTCCGGGC-3′

## Supplementary information

supplementary figs 1,2,3
