# Supplementary material for: Epitope mapping of anti-PGRMC1 antibodies reveals the non-conventional membrane topology of PGRMC1 on the cell surface
Source: Sci Rep. 2019 Jan 24;9:653. doi: 10.1038/s41598-018-37441-6 (PMC6345922; doi:10.1038/s41598-018-37441-6)
Supplement: Supplementary file 1 — supplementary figs 1,2,3 [file 41598_2018_37441_MOESM1_ESM.docx]

**Epitope mapping of anti-PGRMC1 antibodies reveals the non-conventional membrane topology of PGRMC1 on the cell surface**

Ji Yea Kim^1, 3^, So Young Kim^1^, Hong Seo Choi^1^, Sungkwan An^2^, and Chun Jeih Ryu^1^

^1^Institute of Anticancer Medicine Development, Department of Integrative Bioscience and Biotechnology, Sejong University, Seoul, Republic of Korea.

^2^Research Institute for Molecular-Targeted Drugs, Department of Cosmetic Engineering, Konkuk University, Seoul, Republic of Korea.

Correspondence and requests for materials should be addressed to C.J.R (email:[cjryu@sejong.ac.kr](mailto:cjryu@sejong.ac.kr)).

^3^Present address: Research Institute for Molecular-Targeted Drugs, Department of Cosmetic Engineering, Konkuk University, Seoul, Republic of Korea.

**Supplementary Figure 1**

**
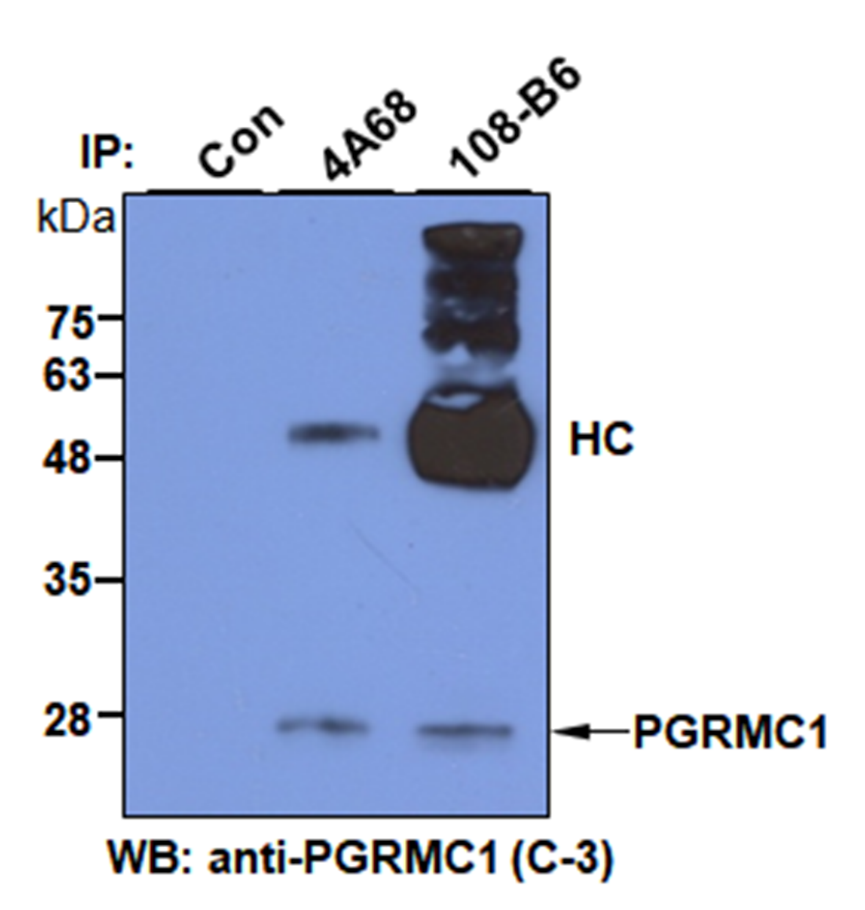
**

**Supplementary Figure 1. Immunoprecipitation and Western blot analysis of 108-B6- and 4A68-reactive PGRMC1.** Cell lysates from NT-2/D1 were subjected to immunoprecipitation with 108-B6 and 4A68, and the immnuoprecipitates were detected by Western blot analysis with C-3. HC, immunoglobulin heavy chain.

**Supplementary Figure 2**

**
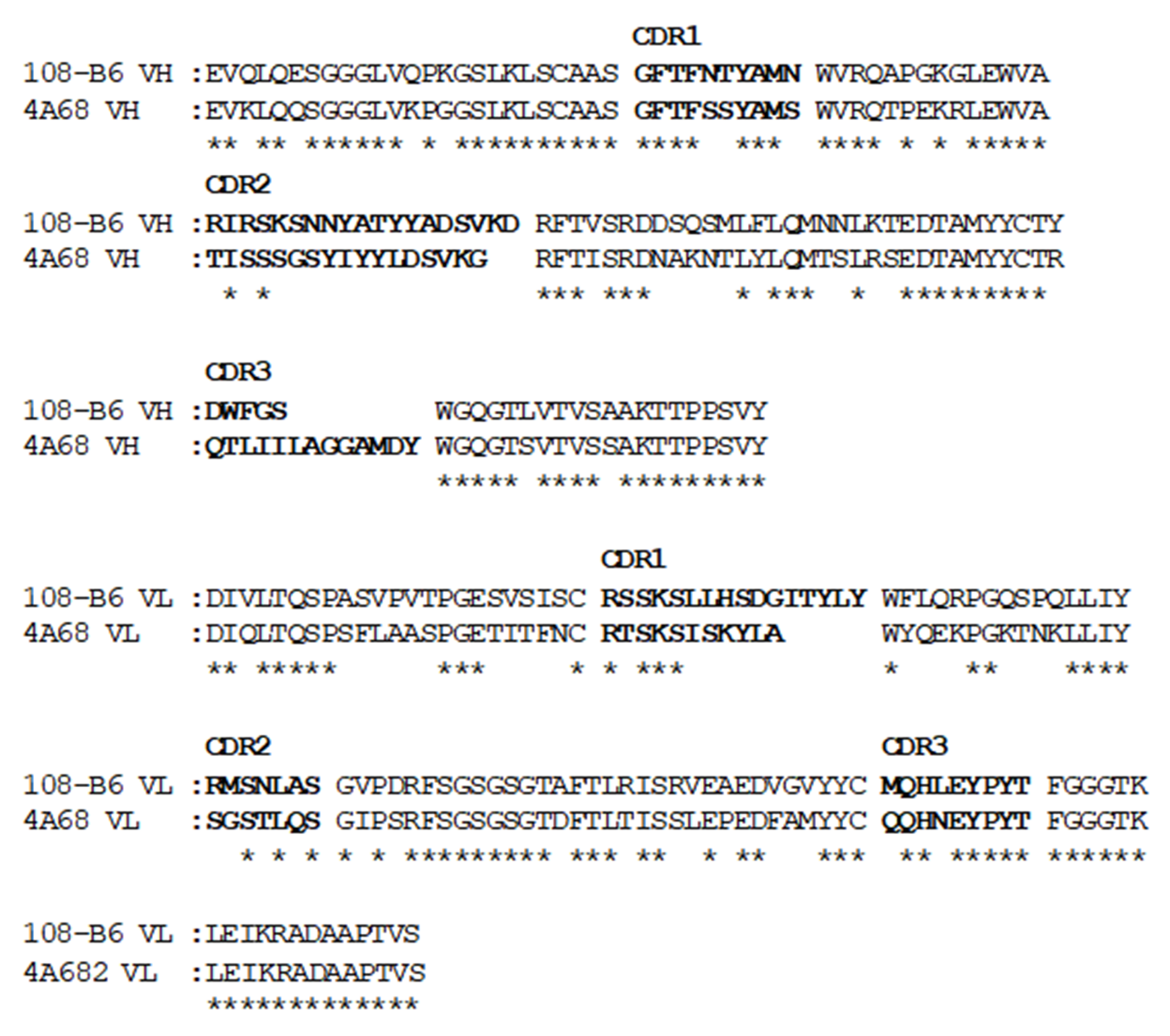
**

**Supplementary Figure 2. Comparison of amino acid sequence of 108-B6 and 4A68 variable regions.** The amino acid sequences of the CDR of the two MAbs are highlighted, and the same amino-acid residues between 108-B6 and 4A68 were marked with asterisk.

**Supplementary Fig. 3**


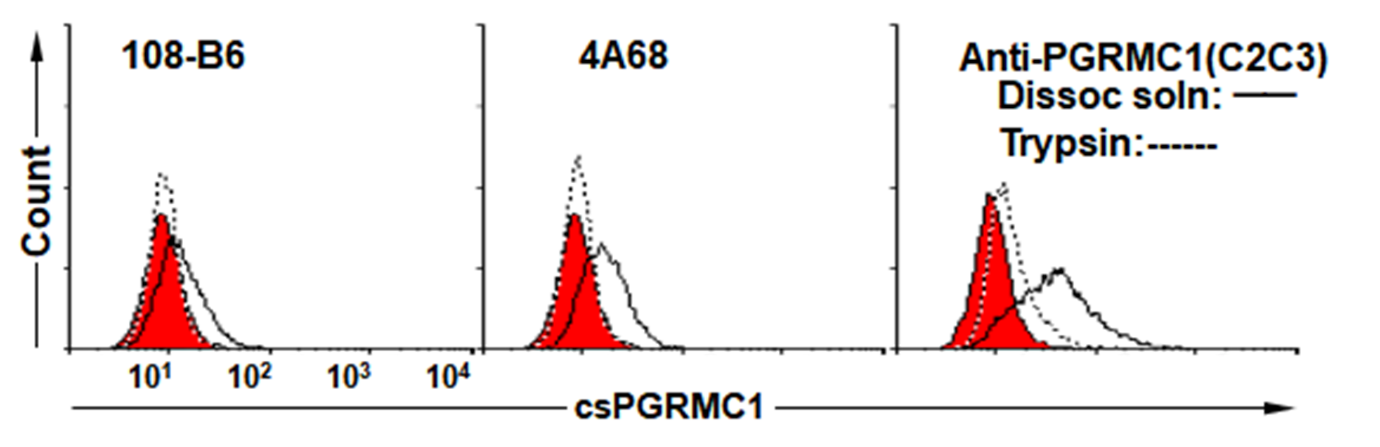


**Supplementary Figure 3.** Flow cytometric analysis of A549 cells with 108-B6, 4A68 and anti-PGRMC1 antibody (C2C3) after detachment of cells with trypsin or enzyme-free dissociation solution. Red populations indicate fluorescence-conjugated secondary antibody staining as controls.
